# Risk factors for the formation of double-contour sign and tophi in gout

**DOI:** 10.1186/s13018-019-1280-0

**Published:** 2019-07-29

**Authors:** Chao Sun, Xuan Qi, Yu Tian, Lixia Gao, Hongtao Jin, Huifang Guo

**Affiliations:** Department of Rheumatology and Immunology, The Second Hospital of Hebei Medical University, No. 215 Heping West Road, Xinhua District, Shijiazhuang, 050000 Hebei Province China

**Keywords:** Gout, Ultrasound, Double-contour sign, Tophi, Risk factor, Questionnaire

## Abstract

**Background:**

This study aimed to confirm the diagnostic accuracy of ultrasound (US) on gout and explore the potential risk factors for double-contour sign and tophi formation in gout patients.

**Methods:**

The US analyses were performed on all knee, ankle, and first metatarsophalangeal (MTP 1) joints to reveal the type and location of lesions. While a questionnaire and blood biochemical index were used to explore the potential risk factors for double-contour sign and tophi in gout, the SPSS17.0 software was used for statistical analysis in the present study.

**Results:**

Totally, 117 gout patients with 702 joints (38 lesions in knee joint, 93 lesions in ankle joint, and 112 lesions in MTP 1 joint) were enrolled in current analyses. Double-contour sign and joint effusion were the two most outstanding lesion manifestations in knee joints and ankle joints. Tophi and double-contour sign were the two most outstanding lesion manifestations in TMP 1 joints. Moreover, factors including uric acid (UA) level and the highest blood UA were potential risk factors of the double-contour sign, while age and history of US were potential risk factors for tophi.

**Conclusion:**

US was effective on the joints of gout patients. There was US sensitivity for tophi and double-contour sign in MTP 1 joints. The double-contour sign was a potential specific manifestation in knee joints and ankle joints. Furthermore, UA and highest blood UA level were potential risk factors for double-contour sign, while age and US history were potential risk factors for tophi.

## Introduction

Gout is an inflammatory disorder characterized by hyperuricemia and the deposition of monosodium urate (MSU) crystals [1]. It is due to elevated levels of uric acid (UA) in the blood [2]. A high level UA accumulation in joints, tendons, and surrounding tissues can induce episodic gout flares, gouty arthropathy, and tophi formation [3]. Gout affects about 2% of the Western population at some point in their lives [4]. As the most common cause of inflammatory arthritis, gout has already caused a great social burden to human in recent decades [5]. Thus, it is necessary to develop novel strategies for gout treatment.

The investigation of useful risk factors is essential for gout treatment [6]. Epidemic study shows that hypertension, renal insufficiency, hypertriglyceridemia, hypercholesterolemia, hyperuricemia, diabetes, obesity, and early menopause are all higher risk for gout [7, 8]. Actually, the accurate diagnosis is critical for revealing appropriate risk factors of gout [9, 10].In clinical practice, various strategies have been successfully used to detect gout including ultrasonography (US), magnetic resonance imaging (MRI), computed tomography (CT), and X-ray [11, 12]. However, the differential diagnosis between gout and other causes of arthritis can be challenging [13]. Owing to these limitations, recent study shows that the high frequency US has higher diagnostic coincidence efficiency in gout tophus than those of X-ray, CT, and MRI [14]. Based on the US detection, the joint and tendon subclinical involvement are proved to be risk factors of gouty arthritis [15]. US double-contour sign is a specific manifestation of urate deposition in gouty arthritis [16, 17]. The American College of Rheumatology (ACR) and European League Against Rheumatism (EULAR)-gout have already clarified the association between US and double-contour sign [18]. Zhu et al. indicated that double-contour sign increased the sensitivity of sonography for detection of urate deposits in gout [19]. Based on an US pilot study in daily clinical practice, Slot et al. has demonstrated that the double-contour sign is a consistent finding in MTP joints in gout patients [20]. Despite of that, as a deposit of UA crystals, tophi is an outcome measure for chronic gout [21]. The development of gouty tophi can limit joint function and cause bone destruction, leading to noticeable disabilities, especially when gout cannot successfully be treated [22]. Thus, the prediagnosis of clinical sign including double-contour or tophi is important for gout therapy [23]. Although double-contour sign and tophi are the two reliable evidence for gout formation under US detection [24], little is known with the independent predictive risk factors for these evidence. Thus, an investigation based on US detection to explore the potential risk factors for double-contour sign and tophi formation in gout patients is needed.

Based on a newly designed questionnaire and US investigation, the present study aimed at investigating the risk factors for double-contour sign and tophi formation in gout patients. Meanwhile, the diagnostic accuracy of US on gout patients was further confirmed. By revealing the potential factors affecting the deposition of urate, we hoped to enhance the prediagnosis rate of gout in clinical practice.

## Methods

### Patients

Between September 2015 and September 2016, patients with gout who present to the rheumatology department of the Second Hospital of Hebei Medical University were recruited in the present study. The inclusion criteria were (1) primary gout arthritis and (2) in accordance with gout diagnostic criteria of the American Society for Rheumatology (ACR). All the patients conformed to the criteria for the classification of the acute arthritis of primary gout [25]. Patients with rheumatoid arthritis, reactive arthritis, psoriatic arthritis, spinal arthritis, or other inflammatory arthritis were excluded. Ethical approval for the present study was obtained from the Second Hospital of Hebei Medical University ethics committee. Meanwhile, the informed consent was obtained from all participants.

### Questionnaire index

All gout patients were investigated with a unified questionnaire. The questionnaire parameters included (1) gender, age, height, and weight; (2) the duration of disease; (3) the frequency of gout attacks over the past 1 year; (4) the highest blood UA level, the usual blood UA level, and the detection frequency of blood UA; (5) usual eating habits; (6) medication history; (7) the history of uric acid-lowering drugs; (8) complications (such as coronary heart disease, diabetes, chronic kidney disease, hyperlipidemia); (9) the history of known tophi, kidney stones, or articular US; (10) whether there is a long-term treatment plan for gout; (11) knowledge of gout; and (12) knowledge of the high purine food. Then, the body mass index (BMI) was calculated by a same physician. The BMI is defined as the body mass divided by the square of the body height and is universally expressed in kg/m^2^ [26]. In the present study, the BMI of 18.5–24 kg/m^2^ represented normal, 24–28 kg/m^2^ represented overweight, and greater than 28 kg/m^2^ was considered as obese.

### Biochemical index analysis

A total of 3 ml fasting venous blood was obtained from all participants and then was analyzed using the Roche automatic biochemical analyzer (cobas 8000, Roche Diagnostics Products (Shanghai) Co., Ltd.). The blood urea nitrogen (BUA), creatinine (CREA), and UA were detected using Berthelot’s enzymic colorimetric method [27–29].The total cholesterol (TC) was detected by HMMPS method (cholesterol oxidase) based on total cholesterol assay kit (YZB/JAP 1794-2008, Wako Pure Chemical Industries, Ltd.). The total triglycerides (TG) was detected by glycerine phosphate oxidase-peroxidase (GPO-PAP) method based on TG assay kit (TR7971, Randox Laboratories Ltd). All the operation of the assay kits were strictly according to the manufacturer’s instruction.

### Ultrasound investigation

The representative US images of each individual elementary lesion presented in the longitudinal and transverse scans from each patient were collected to observe the pathological changes of joint effusion, synovial hyperplasia, synovitis, bone erosion, gout, and double-contour sign. The detailed US examinations were as follows: knee (hyaline cartilage of the femoral condyles; patellar tendon, including both proximal and distal insertion; femoral bone profile; operated with 4–13 MHz linear array probe), ankle (Achilles tendon), and foot (first metatarsophalangeal joint (MTP 1) for hyaline cartilage, bone profile, periarticular tissue). These anatomical areas were selected because of their accessibility by US and their frequent involvement in patients with gout. Based on the full digital color Doppler ultrasound diagnostic instrument (ESAOTE MyLab 90, Genoa, Italy), all the US investigations were performed by the same doctor who had received a formal musculoskeletal US training.

### Statistical analysis

The SPSS17.0 software (SPSS, Inc., Chicago, IL, USA) was used for statistical analysis in the present study. The distribution of the quantitative data was represented by mean ± standard deviation. The normality test was performed by the Shapiro-Wilk method. The means in two groups were compared with *t* test if the data was conformed to normal distribution; if not, the Mann-Whitney *U* test was used [30]. The differences of qualitative data between groups were compared with the chi-square test. The analyses of risk factors for double-contour sign and tophi formation were performed using logistic binary regression. Bilateral *P* < 0.05 was considered as statistically significant.

## Results

### Baseline characteristics

A total of 117 gout patients were enrolled in this study (114 males and 3 females, average age 40.32 ± 11.93 years). The average BMI was 28.34 ± 5.38 kg/m^2^. There were 81 patients with acute stage and 36 patients with intermittent period. The US detection was performed on a total of 234 knee joints, 234 ankle joints, and 234 MTP 1 joints (Table 1). The results showed that there were 38 lesions (16.2% of 234 knees) in knee joints, 93 lesions (39.7% of 234 ankles) in ankle joints, and 112 lesions (47.9% of 234 MTP 1) in MTP 1 joints.

**Table 1:** The number of abnormal joints in gout patients under ultrasonic examination

Area	Total joints	Lesions		The percentage of lesions in total joints (%)	Chi-square value	*P* value
		Right side	Left side			
Knees	234	22 (18.8%)	16 (13.7%)	16.2	19.983	< 0.001
Ankles	234	50 (42.7%)	43 (36.8%)	39.7	11.174	0.001
MTP 1	234	56 (47.9%)	56 (47.9%)	47.9	3.706	0.054

*MTP 1* first metatarsophalangeal joint

### Lesions examination of joints

**Fig. 1:**
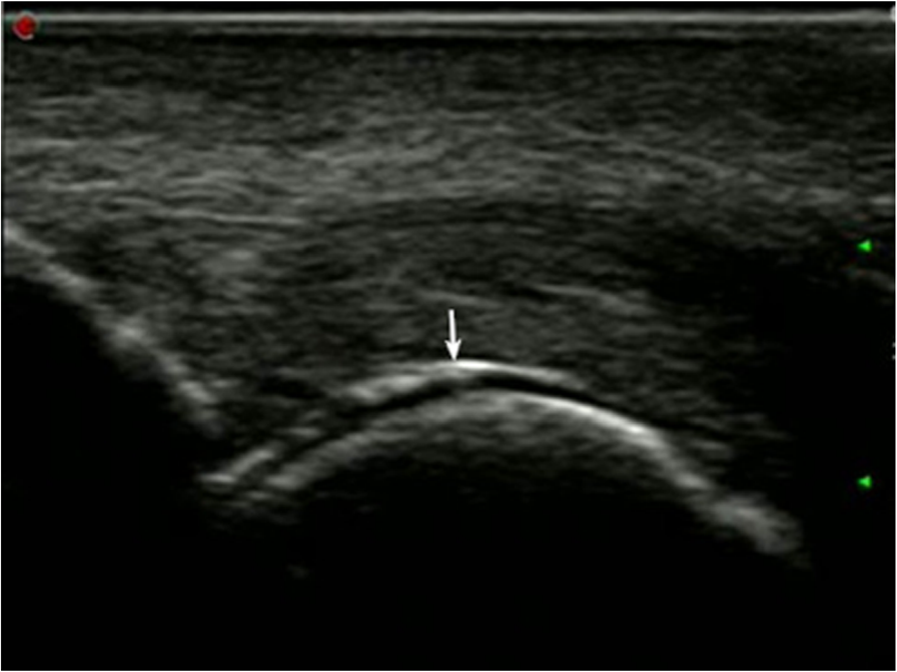
The ultrasound image for double-contour sign in gout patients. The white arrow represented the signal of double-contour sign in gout patients

**Fig. 2:**
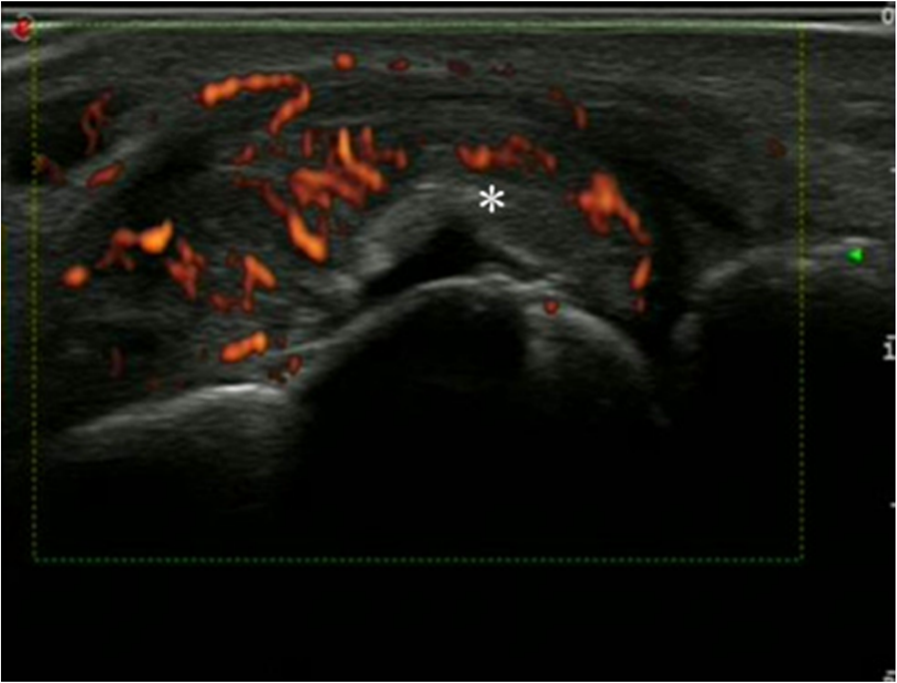
The ultrasound image for tophi in gout patients. The red signal represented the tophi in gout patients

The pathological manifestations of all kinds of joint (knees, ankles, and MTP 1) were explored by US examination (Table 2). The results showed that double-contour sign (30 joints) and joint effusion (17 joints) were the two most outstanding manifestations of knees in gout patients. Meanwhile, double-contour sign (44 joints) and joint effusion (42 joints) were the two most outstanding manifestations of ankles in gout patients. Furthermore, the tophi (78 joints) and double-contour sign (64 joints) were the two most outstanding manifestations of MTP 1 in gout patients. The representative US images for double-contour sign and tophi are shown in Figs. 1 and 2, respectively.

**Table 2:** The pathological manifestations of knees, ankles and MTP 1 joints in gout patients

Area	Hypodermic edema	Joint effusion	Tenosynovitis	Synovial hyperplasia	Synovitis	Tophi	Double-contour sign	Bone erosion	Tendon sheath effusion	Crystal deposition
Right knee	0	11	0	0	1	7	16	0	0	0
Left knee	0	6	0	0	1	4	14	0	0	0
Right ankle	18	20	3	2	6	6	23	0	0	0
Left ankle	17	22	1	1	4	7	21	0	1	1
Right MTP 1	0	1	0	1	15	36	33	17	0	3
Left MTP 1	1	2	0	0	11	42	31	19	0	1

*MTP 1* first metatarsophalangeal joint

### Risk factors analysis of double-contour sign

All the parameters in the current questionnaire were included in the risk factor investigation. The significance test of double-contour sign showed that UA level (*P* < 0.01), peak blood UA (*P* < 0.01), and disease duration (*P* < 0.01) were associated with the occurrence of double-contour sign (Table 3).Then, the logistic regression analysis of risk factors was performed on double-contour sign based on the significance test. The results showed that UA (*P* = 0.011; OR = 1.006; 95% CI = 1.001–1.010), highest blood UA (*P* = 0.014; OR = 7.570; 95% CI = 1.511–37.930), drug intervention history in the intermittent period (*P* = 0.041; OR = 3.468; 95% CI = 1.036–5.876), and history of US (*P* = 0.039; OR = 8.234; 95% CI = 1.117–60.710) were potential independent risk factors for the double-contour sign (Table 4).

**Table 3:** The significance test of different parameters on double-contour sign in gout patients

Parameters	Groups	Without double-contour sign	With double-contour sign	P value
Age*		37.510 ± 11.709	42.640 ± 11.694	0.020
BUN (mmol/L)		5.094 ± 1.461	5.368 ± 1.719	0.458
CREA (μmol/L)		75.672 ± 17.683	78.345 ± 19.149	0.429
UA (μmol/L)*		443.640 ± 114.603	518.120 ± 131.620	0.002
TC (mmol/L)		4.781 ± 1.178	4.823 ± 1.450	0.646
TG (mmol/L)		2.049 ± 1.401	2.179 ± 1.644	0.916
FBG (mmol/L)		5.217 ± 0.708	5.315 ± 1.004	0.511
Sex	Female	3	0	0.180
	Male	50	64	
BMI	Normal	7	11	0.598
	Overweight	22	21	
	Obesity	24	32	
Duration	≤ 1 year	23	4	< 0.001
	1–5 years	21	35	
	≥ 5 years	9	25	
Gout attack in 1 year	0–2 times	29	13	< 0.001
	3–6 times	20	27	
	7–12 times	4	24	
Peak blood UA	421–539	19	5	< 0.001
	≥ 540	34	59	
UA level	≤ 421	13	3	< 0.001
	421–539	20	15	
	≥ 540	20	46	
Frequency of UA or renal function examination	Regularly checked	13	5	0.053
	Occasionally checked	18	26	
	Check only when gout attacks	22	31	
Eating habits	Strict diet	14	17	0.967
	Avoid the high purine diet as much as possible, but not strictly controlled	23	29	
	No control over diet	16	18	
Medication during the interval	Insist on taking	15	5	0.009
	Without medications	29	41	
	Occasional medications	8	17	
Hypertensive	No	43	45	0.177
	Yes	10	19	
CHD	No	52	60	0.482
	Yes	1	4	
Diabetes	No	51	62	1.000
	Yes	2	2	
CKD	No	49	63	0.257
	Yes	4	1	
Other diseases	No	34	38	0.597
	Yes	19	26	
Hyperlipidemia	No	48	61	0.519
	Yes	5	3	
Kidney stones	Yes	14	11	0.277
	No	29	38	
Tophi	Yes	10	16	0.086
	No	19	12	
Ever done a joint US	Done	9	3	0.029
	Never done	43	60	
Whether there is a long-term treatment plan for gout	Yes	21	15	0.059
	No	32	49	
Understand gout	Understand	13	11	0.516
	A little	27	34	
	Not understand	12	19	
Knowledge of high purine food	Fully understand	18	14	0.334
	A little	24	34	
	Unknown	10	15	

*UA* uric acid, *CHD* coronary heart disease, *CKD* chronic kidney disease, *US* ultrasound. *P* < 0.05 was considered as significantly different

^*^t test

**Table 4:** Logistic regression analysis of risk factors for double-contour sign in gout patients

Variables	*P*	OR	95% CI
Age	0.067	1.051	0.997–1.108
UA (μmol/L)	0.011	1.006	1.001–1.010
Duration of gout	0.062	2.322	0.958–5.625
Gout attacks over the past 1 year	0.067	2.063	0.951–4.474
The highest UA level	0.014	7.570	1.511–37.930
Peak blood UA level	0.937	0.962	0.366–2.529
Drug intervention history in the intermittent period	0.041	2.468	1.036–5.876
History of US	0.039	8.234	1.117–60.710
Constants	< 0.001		

*UA* uric acid, *OR* odds ratio, *CI* confidence interval. *P* < 0.05 was considered as significantly different

### Risk factors analysis of tophi

The significance test of double-contour sign and tophi is listed in Table 5. The results showed that the UA level (*P* = 0.007), frequency of UA or renal function examination (*P* = 0.002), and ever done a joint US (*P* < 0.01) were associated with the occurrence of tophi. The logistic regression analysis of risk factors was performed on tophi in gout patients based on the significance test. The risk factor investigation showed that age (mean age of patients with tophi 42.640 ± 12.112; mean age of patients without tophi 36.980 ± 10.940; *P* = 0.008; OR = 1.070; 95% CI = 1.018–1.124) and history of US (*P* = 0.006; OR = 26.801; 95% CI = 2.529–284.051) were potential independent risk factors for tophi (Table 6).

**Table 5:** The significance test of different parameters on tophi in gout patients

Parameters	Groups	Without double-contour sign	With double-contour sign	*P* value
Age*		36.980 ± 10.940	42.640 ± 12.112	0.011
BUN (mmol/L)		5.043 ± 1.380	5.383 ± 1.743	0.372
CREA (μmol/L)		77.488 ± 22.947	76.888 ± 14.758	0.331
UA (μmol/L)*		478.167 ± 145.745	488.704 ± 117.178	0.678
TC (mmol/L)		4.828 ± 1.400	4.789 ± 1.296	0.618
TG (mmol/L)		1.991 ± 1.578	2.208 ± 1.515	0.125
FBG (mmol/L)		5.225 ± 0.674	5.303 ± 1.002	0.751
Sex	Female	1	2	1.000
	Male	47	67	
BMI	Normal	10	8	0.233
	Overweight	19	24	
	Obesity	19	37	
Duration	≤ 1 year	16	11	0.059
	1–5 years	22	34	
	≥ 5 years	10	24	
Gout attack in 1 year	0–2 times	24	18	0.019
	3–6 times	17	30	
	7–12 times	7	21	
Peak blood UA	421–539	11	13	0.591
	≥ 540	37	56	
UA level	≤ 421	12	4	0.007
	421–539	15	20	
	≥ 540	21	45	
Frequency of UA or renal function examination	Regularly checked	14	4	0.002
	Occasionally checked	15	29	
	Check only when gout attacks	18	35	
Eating habits	Strict diet	16	15	0.286
	Avoid the high purine diet as much as possible, but not strictly controlled	21	31	
	No control over diet	11	23	
Medication during the interval	Insist on taking	14	6	0.010
	Without medications	24	46	
	Occasional medications	8	17	
Hypertensive	No	35	53	0.631
	Yes	13	16	
CHD	No	48	64	0.149
	Yes	0	5	
Diabetes	No	47	66	0.884
	Yes	1	3	
CKD	No	45	67	0.677
	Yes	3	2	
Other diseases	No	29	43	0.835
	Yes	19	26	
Hyperlipidemia	No	43	66	0.364
	Yes	5	3	
Kidney stones	Yes	12	13	0.782
	No	30	37	
Tophi	Yes	10	16	0.140
	No	18	13	
Ever done a joint US	Done	11	1	< 0.001
	Never done	37	66	
Whether there is a long-term treatment plan for gout	Yes	21	15	0.011
	No	27	54	
Understand gout	Understand	14	10	0.050
	A little	26	35	
	Not understand	8	23	
Knowledge of high purine food	Fully understand	17	15	0.083
	A little	25	33	
	Unknown	6	19	

*UA* uric acid, *CHD* coronary heart disease, *CKD* chronic kidney disease, *US* ultrasound. *P* < 0.05 was considered as significantly different

*t test

**Table 6:** Logistic regression analysis of risk factors for tophi in gout patients

Variables	*P*	OR	95% CI
Age	0.008	1.070	1.018–1.124
Gout attacks over the past 1 year	0.385	1.332	0.697–2.548
Drug intervention history in the intermittent period	0.422	1.367	0.638–2.928
Joints US history	0.006	26.801	2.529–284.051
Whether there is a long-term treatment plan	0.512	1.414	0.502–3.982
Blood UA level	0.068	2.111	0.946–4.712
Frequency of UA or renal function test	0.330	1.426	0.699–2.910
Constants	< 0.001		

*UA* uric acid, *OR* odds ratio, *CI* confidence interval. *P* < 0.05 was considered as significantly different

## Discussion

Gout is characterized with deposition of urate including double-contour sign and tophi [31]. The risk factors that participate in the process of urate crystal formation are vital for the prediagnosis and treatment of gout [32]. To reveal the US diagnostic effect and potential risk factors affecting the deposition of urate, a study was performed based on US and questionnaire investigation. Totally, 117 gout patients with 702 joints were enrolled in current analyses. In those 702 joints, there were 38 lesions (16.2% of 234 knees) in knee joints, 93 lesions (39.7% of 234 ankles) in ankle joints, and 112 lesions (47.9% of 234 MTP 1) in MTP 1 joints. Double-contour sign and joint effusion were the two most outstanding lesion manifestations in knee joints and ankle joints. Meanwhile, tophi and double-contour sign were two most outstanding lesion manifestations in TMP 1 joints. Based on the questionnaire and blood biochemical index detection, the logistic regression analyses showed that UA, highest blood UA, drug intervention history in the intermittent period, and history of US were potential risk factors of the double-contour sign, while age and history of US were potential risk factors for tophi.

Urate deposition is closely related to the structural joint damage in gout patients [33]. US can reflect the concurrent validity of urate deposition change [34]. Naredo et al. indicated that US bilateral assessment might be valid for diagnosing gout with acceptable sensitivity and specificity [35]. Due to the benefits of safe, non-invasive, free of ionizing radiation, less expensive, and multiple-target assessment in real time, US is the optimal tool for urate deposition monitoring in gout patients [36]. In this study, the US detection rate of joint lesions in 234 knee joints, 234 ankle joints, and 234 MTP 1 joints was 16.2%, 9.7%, and 47.9%, respectively. These results showed that US could reveal lesions in all three kinds of joints in gout patients. Interestingly, the occurrence rate of lesion in MTP 1 joints was significantly higher than that in knee joints and ankle joints in the current study. Pineda et al. showed that the double-contour sign was found in almost 25% of MTP 1 joints (higher than any other kinds of joints) of gout patients [37]. Previous studies indicate that the double-contour sign and tophi are the two classical manifestations of urate deposition in joints of gout patients [16, 17, 38]. However, based on a meta-analysis of the diagnostic accuracy for US, Young et al. showed that US signs of tophi and the double-contour sign were not sensitive in gout patients [39]. Singh and Dalbeth even doubt that the double-contour sign was not specific for gout but for calcium pyrophosphate crystal deposition or other arthritis [17]. Thus, although US is optimal tool for urate deposition monitoring, the US diagnostic sensitivity and specificity for tophi and the double-contour sign in gout patients is controversial. In the present study, US examination showed that the double-contour sign was one of the most outstanding lesion manifestations in both knee joints and ankle joints, while the tophi and double-contour sign were the two most outstanding lesion manifestations in TMP 1 joints. Based on those results, we speculated that there might be an US sensitivity for tophi and double-contour sign in MTP 1 joints. Furthermore, the double-contour sign might be the specific manifestation in knee joints and ankle joints, which was different from the results of Singh and Dalbeth [17]. The reason for this difference might be the larger sample size of knee and ankle joints enrolled in the present study. However, a further investigation is needed to confirm the results obtained in this study.

In gout patients, UA level, double-contour sign, and tophi as well as ankle musculoskeletal examination have high diagnostic value in clinical practice [40]. The interaction between UA level and other risk factors in the development of gout has been proved in the previous study [41]. Although the increased UA level is a major risk factor for gout, Kumar et al. showed that serum UA level did not confirm or excluded gout; many people did not develop gout, and during acute attacks, serum levels might be normal [42]. A biochemical analyses in previous report showed that the UA concentration in the knee joint of a gout patient was consistently less than 5 mg/dL (297.6 μmol/L), but the US confirmed a resemblance of the double-contour sign typical of UA deposits [43]. Moreover, many researchers believe that serum UA levels cannot be considered a sensitive marker for double-contour sign during the diagnosis of gout [44, 45]. Actually, the logistic regression analyses in this study showed that UA and highest blood UA were both risk factors for double-contour sign. Furthermore, recent data suggest that the prevalence of gout is increased with age both in men and women [46]. A previous logistic regression analysis shows that age is one of the risk factors associated with tophi formation in gout [23]. A meta-analysis of cigarette smoking on gout occurrence shows that age is an influence factor for the occurrence of gout [47]. Although tophi are an important manifestation in gout, the study focused on relation between age and tophi formation is rare. In this study, the occurrence of tophi formation in low age group (mean age 36.980 ± 10.940) was significantly lower than the high age group (mean age 42.640 ± 12.112). Thus, based on the logistic regression investigation, we speculated that the risk of tophi formation might increase with the age in gout patients. Interestingly, the risk factor analyses in the current study showed that the patients who had US history might have a lower occurrence of tophi formation than patients without US history. We speculated that a potential threptic effect of US operation or patient itself raises awareness of the prevention for pre-existing diseases might be the reasons. Unfortunately, there is no such report on US history decreasing the formation of tophi. Thus, a further investigation to confirm the effect of US history on tophi formation is needed. However, there were still some limitations in the current study such as small sample size and lack of subsequent verification test.

## Conclusions

In conclusion, the diagnostic accuracy of US on the joints of gout patients might be ideal. There might be an US sensitivity for tophi and the double-contour sign in MTP 1 joints, while the double-contour sign might be the specific manifestation in knee joints and ankle joints. Furthermore, UA and peak blood UA level might be the potential risk factors for double-contour sign, while age and US history might be the potential risk factors for tophi in gout.

## Data Availability

Not applicable.

## Abbreviations

ACR: American College of Rheumatology
BMI: Body mass index
BUA: Blood urea nitrogen
CREA: Creatinine
CT: Computed tomography
EULAR: European League Against Rheumatism
GPO-PAP: Glycerine phosphate oxidase-peroxidase
MRI: Magnetic resonance imaging
MSU: Monosodium urate
MTP 1: First metatarsophalangeal joint
TC: total cholesterol
TG: Total triglycerides
UA: Uric acid
US: Ultrasound
